# 10 years later: Time for a change

**Published:** 2019-03

**Authors:** W Ombelet

Back in June 2009 the first issue of “Facts, Views and Vision in Obgyn” (FV&V) was published as an international journal with a new concept in the world of scientific journals.

With the paramount goal of reaching our public and in the scope of a globalized world, we opted to offer an open access journal, rendering the content of each and every of our published issues free and available without any charge to the user or his/her institution, plus allowing the reader to download, copy, distribute, print, search, or link full texts and content of the articles in this journal without asking for prior permission from the publisher or the author.

From the very beginning it was clear that the scope in our papers would be different from that in most scientific journals. Indeed, we were convinced with the promise of offering a unique forum for socio-cultural, ethical, philosophical, health-economical and political debates related to our work as gynaecologists and reproductive health care providers. Considering the facts: original studies, pilot-studies, reviews and PhD summaries became the backbone of our journal. We witnessed the publication of many viewpoints and opinion papers critically commenting the facts provided in the literature and last but-not-least we also published many strong opinions of international key opinion leaders, shedding a visionary dimension to what currently raises interest in the broad medical field of human reproduction. With surprise, we were fascinated to observe that many papers grasped lots of interest in the global perspective of our specialty, thus, reaching our paramount aim.

Thanks to our artistic reviewer, Koen Vanmechelen, FV&V realised a different but very attractive look compared to other scientific journals. Indeed, the confrontation of art and science turned out to be something unique, a cross-breeding at its best.

As a major achievement in the history of our Journal, in 2014, we succeeded to become PubMed-cited. Nowadays the Journal is covered by major indexing services such as PMC (PubMed Central), Pubmed, Web of Science, Google Scholar, BMJ Clinical Evidence and Europe PMC.

During the past 10 years, from 582 submissions, 310 papers have been published. As such, our journal extended an acceptance rate of 53.2 % with more than 20 % of our publications (66/310) being “Opinion and Viewpoint” articles.

After 10 years, we can only be positively surprised about the number of citations we have achieved, a good parameter of the impact of our papers on the actual scientific literature of obstetrics, gynaecology andreproductive health (Ombelet, 2018).

From 2019 onwards, our Journal will be owned by the European Society for Gynaecological Endoscopy (ESGE) in cooperation with the Walking Egg non-profit organization. Hence, it will become the official Journal of the ESGE, with a majority of papers related to the surgical aspects of endoscopic imaging and allied techniques. Another part of the Journal will be creserved to continue publishing selected papers as we did before.

Additionally, from its very beginning our journal has embraced all ObGyn disciplines and placed a major interest in images, art contributions and visionary ideas. Therefore, we believe that FV&V has provided and will continue to provide the perfect scientific environment to initiate the use of video’s, a novelty in our scientific community.

With much satisfaction, we are still associated on our artistic reviewer, Koen Vanmechelen, who is responsible for the very attractive and unique look of our journal. Koen is one of most influential contemporary artists today; finding his inspiration in the wide crossroad of art, science, politics, philosophy and ethics.

Opinion and viewpoint papers will continue to be confronted with his artistic interpretation to further stimulate and encourage discussions in a truly holistic context, thus, adding a unique dimension to our journal.

As the Editor-in-Chief, I look back on 10 exciting years and therefore I would like to thank the editorial board, all the authors and referees who contributed to the journal since 2009. Also, allow me to express my special gratitude to the ‘Universa Press’ team for their highly dedicated work.

It has been an honour and a real pleasure to be part of this exciting project. A bright future for our journal is awaiting us.

Willem Ombelet Editor-in-Chief
